# Comparative proteomic analysis of metronidazole-sensitive and resistant *Trichomonas vaginalis* suggests a novel mode of metronidazole action and resistance

**DOI:** 10.1016/j.ijpddr.2024.100566

**Published:** 2024-09-26

**Authors:** Anna-Lena Mayr, Ana Paunkov, Karin Hummel, Ebrahim Razzazi-Fazeli, David Leitsch

**Affiliations:** a VetCore Facility for Research, University of Veterinary Medicine, Veterinärplatz 1, 1210, Vienna, Austria; b Institute of Specific Prophylaxis and Tropical Medicine, Medical University of Vienna, Kinderspitalgasse 15, 1090, Vienna, Austria

## Abstract

The microaerophilic parasite *Trichomonas vaginalis* occurs worldwide and causes inflammation of the urogenital tract, especially in women. With 156 million infections annually, trichomoniasis is the most prevalent non-viral sexually transmitted disease. Trichomoniasis is treated with 5-nitroimidazoles, especially metronidazole, which are prodrugs that have to be reduced at their nitro group to be activated. Resistance rates to metronidazole have remained comparably low, but they can be higher in certain areas leading to an increase of refractory cases. Metronidazole resistance in *T*. *vaginalis* can develop *in vivo* in clinical isolates, or it can be induced in the laboratory. Both types of resistance share certain characteristics but differ with regard to the dependence of ambient oxygen to become manifest. Although several candidate factors for metronidazole resistance have been described in the past, e.g. pyruvate:ferredoxin oxidoreductase and ferredoxin or thioredoxin reductase, open questions regarding their role in resistance have remained.

In order to address these questions, we performed a proteomic study with metronidazole-sensitive and –resistant laboratory strains, as well as with clinical strains, in order to identify factors causative for resistance. The list of proteins consistently associated with resistance was surprisingly short. Resistant laboratory and clinical strains only shared the downregulation of flavin reductase 1 (FR1), an enzyme previously identified to be involved in resistance. Originally, FR1 was believed to be an oxygen scavenging enzyme, but here we identified it as a ferric iron reductase which produces ferrous iron. Based on this finding and on further experimental evidence as presented herein, we propose a novel mechanism of metronidazole activation which is based on ferrous iron binding to proteins, thereby rendering them susceptible to complex formation with metronidazole. Upon resolution of iron-protein-metronidazole complexes, metronidazole radicals are formed which quickly react with thiols or proteins in the direct vicinity, leading to breaks in the peptide backbone.

## Highlights

• Downregulation of FR1 is the only trait consistently shared by resistant *T. vaginalis*.
• Clinical resistant isolates are also partially resistant under anaerobic conditions.
• FR1 is a ferric reductase.
• A new model of metronidazole action and resistance is proposed.

## Graphical abstract

**Figure:**
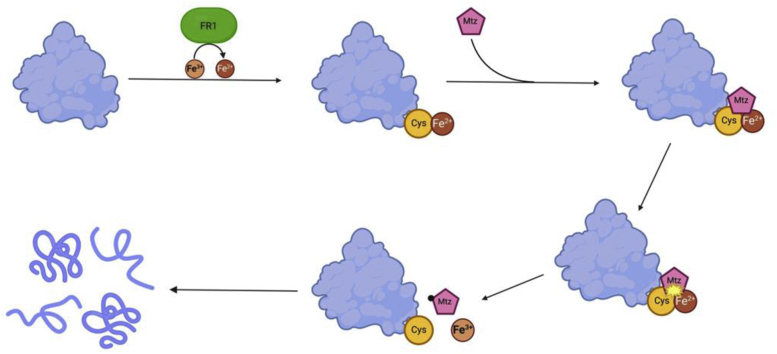

## 1 Introduction

The anaerobic/microaerophilic protozoan *Trichomonas vaginalis* is a human parasite which causes an inflammation of the urogenital tract, commonly termed trichomoniasis. This infection constitutes one of the most frequent sexually transmitted diseases worldwide (Rowley et al., 2019) and mostly affects women. In the majority of cases the symptoms are mild, but they can also be severe and debilitating, including odorous vaginal discharge, pruritus, and pain. Importantly, underlying trichomoniasis increases the likelihood of adverse pregnancy outcomes and predisposes for the contraction of HIV (Masha et al., 2019), the latter a particularly serious health problem in countries where HIV prevalence is high. Since no vaccine is available against *T*. *vaginalis*, the management of trichomoniasis solely relies on drug therapy.

The treatment of trichomoniasis is almost exclusively based on 5-nitroimidazole drugs, mainly metronidazole. Interestingly, metronidazole was specifically developed in 1959 for the treatment of *T*. *vaginalis* infections but it is also effective against most other anaerobic and microaerophilic pathogens (Leitsch, 2019). 5-nitroimidazoles are administered as prodrugs which are intracellularly reduced at the nitro group leading to the formation of toxic intermediates. Quantitative reduction of the nitro group only occurs in the (near) absence of oxygen, rendering it widely safe for organisms with an aerobic metabolism. It has remained unresolved as to which reduction product is the actual toxic agent, but the single e^−^ transfer product, the corresponding 5-nitroradical anion, as well as the two e^−^ transfer product, the corresponding 5-nitrosoimidazole, have toxic effects in an intracellular environment (Leitsch, 2019). In *T*. *vaginalis*, metronidazole derivatives were demonstrated to bind to protein and DNA (Ings et al., 1974). They also decrease intracellular thiol levels and form covalent adducts with a specific subset of proteins (Leitsch et al., 2009), including thioredoxin reductase (TrxR) and several enzymes/proteins that are known to interact with TrxR. In the case of TrxR, covalent adduct formation impairs enzyme activity (Leitsch et al., 2009).

Metronidazole resistance has remained comparably rare throughout the decades, although this varies geographically, and in some regions up to 20% of the *T*. *vaginalis* infections have been reported to be refractory to metronidazole treatment (Upcroft et al., 2009). Importantly, treatment failures are not always caused by nitroimidazole resistance as such because in the majority of cases the administration of higher doses of metronidazole or another 5-nitroimidazole drug approved for the treatment of trichomoniasis in many countries, tinidazole, can clear the parasite (Sobel et al., 2001). Still, true metronidazole/5-nitroimidazole resistance does exist which can complicate the treatment of trichomoniasis considerably because alternative options are missing.

Two different types of metronidazole resistance in *T*. *vaginalis* have been described (Leitsch, 2019): 1., *in vitro* resistance (also termed anaerobic resistance) which is induced in laboratory strains through extended exposure to sublethal doses of metronidazole, and 2., clinical resistance (or aerobic resistance, respectively) which occurs in strains isolated from trichomoniasis patients. The former is believed to be caused by the loss of enzymes which reduce the nitro group of 5-nitroimidazoles and thereby activate the prodrug. These include pyruvate:ferredoxin oxidoreductase (PFOR) and hydrogenosomal malate dehydrogenase (hydMDH), which both can reduce metronidazole via their respective cofactor ferredoxin (Kulda, 1999), and TrxR which is not only a target of 5-nitroimidazoles but can also reduce their nitro group (Leitsch et al., 2009). Whereas the expression of PFOR, hydMDH, and ferredoxin was found to be shut off in *T*. *vaginalis* with *in vitro* resistance (Kulda, 1999), TrxR was found to be inactive due to a lack of FAD cofactor bound to the enzyme (Leitsch et al., 2009). In clinical strains with metronidazole resistance, however, these enzymes are all functional (Kulda, 1999; Leitsch et al., 2012) and resistance was only observed in the presence of oxygen. In accordance, isolates displaying clinical resistance were shown to have impaired oxygen scavenging mechanisms (Yarlett et al., 1986), presumably leading to higher intracellular oxygen concentrations.

In both types of resistance, however, the impairment of flavin reductase activity, exerted by flavin reductase 1 (FR1) (Leitsch et al., 2014), was observed (Leitsch et al., 2009, 2012). This enzyme had originally been identified as an oxygen scavenging enzyme in *T*. *vaginalis* which reduces oxygen to hydrogen peroxide via the cofactor FMN (Linstead and Bradley, 1988) and was later found to be the major source of intracellular hydrogen peroxide formation in *T*. *vaginalis* (Chapman et al., 1999). Consequently, FR1 was considered a key enzyme in the removal of intracellular oxygen in *T*. *vaginalis* and the loss of its oxygen scavenging function a prerequisite for metronidazole resistance, both in *in vitro* resistant and in clinical resistant strains (Leitsch et al., 2014). Importantly, episomal expression of FR1 was shown to re-establish susceptibility to metronidazole in a metronidazole-resistant clinical strain which normally lacks FR1 activity, BRIS/92/STDL/B7268 (Leitsch et al., 2014). However, more recent observations seriously question the notion of FR1 functioning as an oxygen scavenger because in a *T*. *vaginalis* cell line exhibiting early-stage metronidazole resistance, FR1 activity was found to be decreased to 20% of its original level without any consequences on oxygen scavenging capacity (Gehl et al., 2021). Thus, the physiological function of FR1 and its role in metronidazole resistance is currently unclear, rendering the current model of metronidazole resistance untenable.

In the light of this impasse, it was argued that a new approach for the identification of factors which are involved in metronidazole resistance, but which had so far been overlooked was needed. To this end, we performed proteomic analyses with metronidazole-sensitive and –resistant cell lines of the *T*. *vaginalis* strain C1 and compared the resulting proteome profiles with the proteome profile of C1 cells grown under iron-restriction. In a previous study it had been shown that reduction of intracellular iron levels using the transition metal chelator bipyridyl leads to similar physiological changes as observed in *in vitro* metronidazole-resistant *T*. *vaginalis*, i.e. a near to total shut-off of PFOR and hydMDH expression, without, however, impacting metronidazole susceptibility (Leitsch et al., 2009). Thus, we hypothesized that only changes in the protein expression of resistant cells as compared to normal cells which are not observed in bipyridyl-treated *T*. *vaginalis* are truly indicative of metronidazole resistance. We further hypothesized that the same changes responsible for *in vitro* metronidazole resistance would also be identified when using another *T*. *vaginalis* strain and repeated the analysis in strain T1. Finally, we hypothesized that an according set of enzymes/proteins responsible for clinical metronidazole resistance could also be identified when comparing the proteome profiles of metronidazole-sensitive and –resistant clinical strains and performed comparative proteomic analyses with two metronidazole-sensitive and two-resistant clinical isolates.

## 2 Materials and methods

### *2.1* Trichomonas vaginalis *strains and culture*

All *T*. *vaginalis* strains used in this study were grown in TYM medium (trypticase, yeast extract, maltose medium) (Diamond, 1957) and sub-cultured every day or every second day when cell densities were sufficiently high. The following strains were studied: C1 (ATCC 30001), T1 (Jung Hsiang Tai, Institute of Biomedical Sciences, Taipei, Taiwan), G3 (PRA-98), and JH31A#4 (ATCC 30236) are metronidazole-susceptible isolates. CDC085 (ATCC 50143) and BRIS/92/STDL/B7268 are clinical metronidazole-resistant *T*. *vaginalis* isolates. For convenience, JH31A#4 and BRIS/92/STDL/B7268 are referred to as JH31A and B7268 in the text. Strain T1 was a generous gift from Ivan Hrdý from Charles University, Prague.

The highly metronidazole-resistant *in vitro* cell line of C1 had been generated earlier (Leitsch et al., 2009), and the highly resistant cell line of T1 was generated accordingly by exposing cells to increasing concentrations of metronidazole with every subculture, starting from 5 μM. The resistant T1 cell line could be grown in the presence of 1 mM metronidazole after only two weeks of subculture.

For the reduction of intracellular iron levels, the TVC1 (ATCC 30001) cell line was grown for 9 passages with the permeable ferrous iron chelating agent bipyridyl at a concentration of 50 μM each time.

### 2.2 Proteomic analysis

#### 2.2.1 Cell harvest and protein isolation

Samples were prepared as described before (Mayr et al., 2024). For cell harvest, flasks (40 ml culture) were placed on ice for 10 min followed by centrifugation at 900×*g*. Cells were washed three times with 1 mL PBS and finally suspended in 500 μL of ultrapure water. Subsequently, 1.5 mL 13.3% TCA in acetone was added, followed by incubation at −20 °C for 2 h. After incubation, cells were centrifuged (17,500×*g*, 4 °C, 10 min) and the cell pellet was then washed four times with 90 % acetone and subsequently air dried. Finally, proteins in the pellets were dissolved in protein buffer (7 M Urea, 2 M Thiourea, 4 % CHAPS, 1% DTT) and incubated on a Thermomixer at 700 rpm for 30min at 25 °C. The sample was then centrifuged (17,500×*g*, 20 °C, 10 min) to remove insoluble material and the supernatant was stored at −80 °C for proteomic analysis. Pierce 660 nm Protein Assay (Thermo Scientific) was used to determine the protein concentration of the sample.

#### 2.2.2 Protein digest and clean-up

Based on a comparison of protein digestion methods (Mayr et al., 2024), filter-aided sample preparation was used. For this, 30 μg of protein were loaded on a 3 kDa ultrafiltration unit (Pall Corporation) and reduced with 20 mM DTT for 30 min at 37 °C followed by alkylation with 60 mM IAA for 30 min at 25 °C in darkness on the filter. Samples were washed twice with 100 μL 50 mM Tris. Then Trypsin/LysC (Promega) in 50 mM Tris was added to a final enzyme concentration of 1:25 (enzyme: protein w/w) followed by overnight digestion at 37° C. Peptides were eluted with 3 × 50 μL 50 mM Tris. The eluate was acidified with concentrated TFA for a pH < 2. For desalting and clean-up Pierce C18 spin columns (Thermo Scientific) were used. Columns were activated by adding 200 μL 50% acetonitrile twice and centrifuging at 1500×*g* between every step. Then columns were equilibrated with 200 μL 5% ACN/0.5% TFA twice. Samples were loaded onto the columns for desalting and cleaning of the peptides. Subsequently, samples were washed twice with 200 μL 5% ACN, 0.5% TFA and eluted twice with 20 μL 70% ACN, 0.1% TFA. Finally, samples were evaporated to dryness in a vacuum centrifuge and samples were resolubilized in 300 μL 0.1% TFA for LC-MS/MS analysis.

#### 2.2.3 Mass spectrometry and data analysis

Samples were analysed using a nano-HPLC ultimate 3000 RSLC system (Dionex) coupled to a high-resolution Q-Exactive HF Orbitrap mass spectrometer (Thermo). The LC system was equipped with a 5 mm Acclaim PepMap μ-precolumn (300 μm inner diameter, 5 μm particle size, 100 Å pore size) for sample pre-concentration and desalting. All solutions, as well as solvent gradient and MS parameters are the same as recently published (Mayr et al., in 2024). For separation of peptides a 25 cm Acclaim PepMap C18 column (75 μm inner diameter, 2 μm particle size, 100 Å pore size) was used. Samples were injected into the nanoHPLC in technical duplicates. Database search was performed using the Proteome Discoverer Software 2.4.1.15 (Thermo) using the Sequest HT search engine. The digestion enzyme was trypsin with a maximum of two missed cleavages. Carbamidomethylation was set as a fixed modification. Oxidation (M), deamidation (NQ), acetylation (Protein N-term), Met-loss (Protein N-term (M)), Met-loss + acetyl (Protein N-Term (M)) and Gln- > pyro-Glu (Q) were set as variable modifications. Precursor mass tolerance was 10 ppm. Fragment mass tolerance was 0.02 Da. Spectra were searched in the Uniprot *Trichomonas vaginalis* database (tx5722, 51,768 sequences, www.uniprot.org, downloaded on 2020.09.20). The cRAP database was used to filter out common contaminants (www.thegpm.org/crap/). The “Minora feature detector” node was used with a minimum trace length of 5 and a maximum ΔRT of isotope pattern multiplets of 0.2 min in the peak and feature detection. Furthermore, in feature to ID linking, PSM confidence was set to “at least high”. Target decoy analysis was performed by searching a reverse database with a strict FDR of 0.01 and a relaxed FDR of 0.05 at protein and peptide level. Intensity-based-label-free quantification (LFQ), and statistical evaluation of data was carried out as recently described (Mayr et al., in 2024). The mass spectrometry proteomics data have been deposited at the ProteomeXchange Consortium via the PRIDE partner repository (https://www.ebi.ac.uk/training/online/courses/proteomics-an-introduction/proteomics-resources-at-the-ebi/pride/) with the dataset identifier PXD051345.

### 2.3 Metronidazole susceptibility assays

Minimal lethal concentrations (MLCs) were determined in 96-well plates. Metronidazole was serially diluted in TYM medium to a final volume of 100 μL, to which 100 μL of an inoculum containing 10,000 cells was added. Plates were incubated at 37 C° for 48 h in airtight sealed 2.5 L boxes under either microaerophilic conditions (O_2_: 6.2–13.2%, CO_2_: 2.5–9.5%) provided by CampyGen™ (Thermo Scientific) or anaerobic conditions (O_2_: 0%, CO_2_: 18%) provided by Anaerocult® A (Merck). After the incubation, MLCs were determined using light microscopy. The concentration at which cells were no longer motile (indicating cell death) was taken as MLC. Each experiment was repeated at least three times, with technical duplicate runs each time to ensure reproducibility.

### 2.4 Measurement of FR1 activity in T1

FR1 activity in T1 and the resistant T1 cell line was measured in 100 mM potassium phosphate buffer, pH 6.25, as described before (Leitsch et al., 2014). This buffer had been found optimal for FR1 activity.

### 2.5 Measurement of ferric iron reductase activity of FR1

First a calibration curve was made by measuring the absorption of bipyridyl-iron complexes at λ_522_ formed by bipyridyl (400 μM) and known concentrations of ferrous iron sulphate (FeSO_4_) in 100 mM potassium phosphate, pH 6.25. Absorption was measured at 10, 20, 30, 40, 50, and 60 μM of added Fe_2_SO_4_ (four independent measurements at each concentration). Subsequently, reduction of 50 μM ferric iron chloride (FeCl_3_) in the same buffer was measured in the presence of 10 μg mL^−1^ recombinant FR1, 20 μM FMN, and 2 mM NADPH. Recombinant FR1 had been produced in *E*. *coli* BL21 AI cells as described previously (Leitsch et al., 2014). Fe^2+^, freshly formed through reduction of Fe^3+^ by FR1, was bound by bipyridyl and the increase of absorption at λ_522_ was recorded. The obtained values were plotted against the calibration curve using GraphPad Prism 10 software.

### *2.6* *Metronidazole radical formation in* T*.* vaginalis *cell extracts*

100 mL of dense but still growing cultures (approximately 2 × 10^8^ cells) were harvested (900×*g*) and washed once with 1 × PBS. Subsequently, cells were taken up in 1 mL 100 mM potassium phosphate, pH 6.25. Cells were then carefully lysed in a Dounce homogenizer and large organelles, including hydrogenosomes, nuclei, and lysosomes, were removed by centrifugation at 20,000×*g* (4 °C) for 20 min. 400 μl of the supernatant were transferred into 0.5 mL Protein LoBind tubes (Eppendorf). Then NADPH (4 mM), FMN (20 μM), 500 μM FeSO_4_, and 1 mM metronidazole were added and the volume missing up to 500 μl was filled up with 100 mM KPO4, pH 6.25. The reactions were incubated at room temperature for 30 min. Afterwards the supernatants were transferred into 2 mL tubes (Eppendorf) and 1.5 mL of 13.3% TCA in acetone was added to precipitate proteins. Precipitates were spun off (20,000×*g* for 20 min at 4 °C) and washed twice in 90% acetone. Finally, pellets were dried, and proteins were resolubilized in 2DE sample buffer (7 M Urea, 2 M Thiourea, 4% CHAPS, 1% DTT). Insoluble matter was subsequently removed by centrifugation (20,000×*g* for 20 min at 20 °C).

### 2.7 2D gel electrophoresis

2D gel electrophoresis was performed as described before (Leitsch et al., 2005, 2009, 2012). Briefly, 400 μg of protein in 2DE sample buffer were used for rehydration of 17 cm immobilized pH gradient (IPG) strips in the pH range of 5–8 (Biorad). Isolelectric focusing was performed in a Protean IEF cell (Biorad) applying the following program: rehydration (50 V, 12 h), 150 V (rapid slope, 1 h), 300 V (rapid slope, 1 h), 2000 V (linear slope, 1 h), 5000 V (linear slope, 2 h), 10,000 V (rapid slope, 7 h). Afterwards strips were equilibrated for gel electrophoresis and SDS-PAGE was performed in Protean II Xi cells (Biorad). Gels were stained with Coomassie Brilliant R.

## 3 Results

### *3.1* *Proteomic analysis of metronidazole-sensitive, metronidazole-resistant and bipyridyl-treated* T*.* vaginalis *C1*

**Fig. 1:**
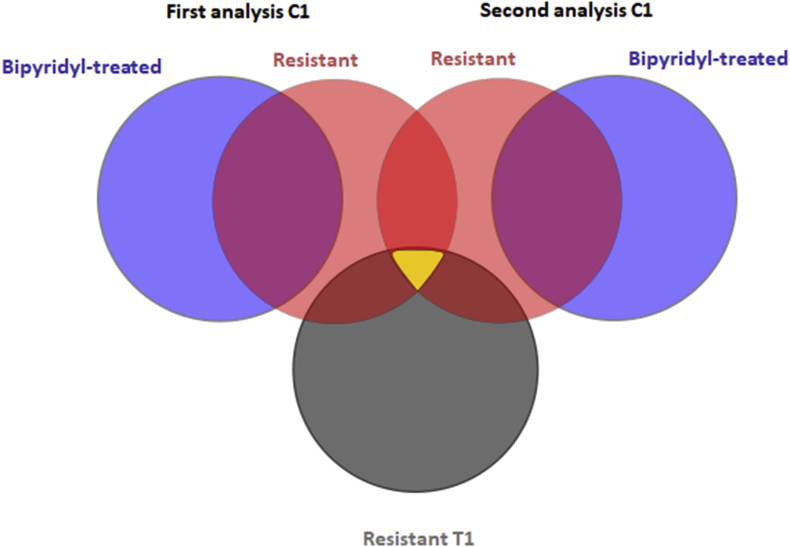
Strategy of the proteomic analysis. In two independent analyses the proteome profiles of control *T*. *vaginalis* C1, 9 × 50 μM bipyridyl-treated C1, and highly *in vitro* resistant C1 were compared. Only proteins that were found to be differentially up- or downregulated twice in resistant C1 but not bipyridyl-treated C1 were matched to the proteins differentially expressed in highly *in vitro* resistant T1 vs. control T1. The resulting subset of proteins (highlighted in yellow) was considered as specific for *in vitro* metronidazole resistance in *T*. *vaginalis* (Table 4). (For interpretation of the references to colour in this figure legend, the reader is referred to the Web version of this article.)

In order to identify proteins which are specifically differentially expressed in metronidazole-resistant cells, we compared the proteome profiles of wildtype *T*. *vaginalis* C1 cells, a highly metronidazole-resistant C1 derivative cell line generated in an earlier study (Leitsch et al., 2009), and of C1 cells cultured nine times in succession in the presence of 50 μM bipyridyl, a ferrous iron chelator which can permeate cell membranes. The extended cultivation of C1 cells with bipyridyl had been found (Leitsch et al., 2009) to lead to a near-to-total shut-off of PFOR and hydMDH, two enzymes supposedly involved in metronidazole activation. We hypothesized that metronidazole-resistant cells downregulate iron uptake and the expression of iron-dependent enzymes, thereby mimicking the changes induced by bipyridyl to a certain extent. However, as bipyridyl-treated cells remain fully susceptible to metronidazole (Table 1), we interpreted all changes in protein expression shared by bipyridyl-treated cells and metronidazole-resistant cells as compared to wildtype cells as not being causative for metronidazole resistance. The proteome profiles of the cells were obtained by performing nano-HPLC-MS/MS and subsequent data analysis with whole cell protein preparations of all three cell lines, i.e. C1 wildtype, C1 resistant, and C1 bipyridyl-treated (Fig. 1). Three biological replicates of each strain, which in turn were divided into two technical duplicates each, were analysed. In order to further enhance reproducibility, the whole analysis was repeated at a later time point and only the proteins featuring as differentially expressed in both data sets were taken into account. Changes which occurred only once were discarded as resulting from natural or technical variation. The total number of proteins detected in the first analysis was 3312, and in the second analysis 3464. This numbers are very close to those reported previously by others (Lin et al., 2020).

**Table 1:** MIC of metronidazole in *T*. *vaginalis* C1. Measurements were performed at least three times in technical duplicates.

Cell line	MIC [μM]
C1, normal	2.342; 9.375; 9.375; 9.375
C1, 9 × subcultured with 50 μM bipyridyl	9.375; 9.375; 9.375
C1, high level metronidazole resistance	4800; 4800; 4800

In order to validate our approach, we compared our results with those by others from an earlier study in which the proteomes of hydrogenosomes from untreated and bipyridyl-treated cells had been compared (Beltrán et al., 2013). The reduction of intracellular iron levels had also been achieved by cultivation in the presence of bipyridyl (10 subcultures with 70 μM bipyridyl). Of the 58 hydrogenosomal proteins found to be differentially expressed in the aforementioned study, we found 36 proteins up- or downregulated with the same tendency in at least one of the two courses of proteomic analysis of our bipyridyl-treated cells (Supplementary Table 1). Another nine proteins were also found in one of our two data sets of bipyridyl-treated cells, but were either not differentially expressed or differentially expressed with opposing tendency, respectively; and finally 13 proteins found by Beltrán et al. were absent from our data sets. Thus, 8 out of 10 proteins found in the earlier study, including also small ones like ferredoxins, were also present in our data sets and more than 6 out of 10 proteins were found to be differentially expressed with the same tendency. We interpreted this high congruence as corroborative evidence for the validity and robustness of our methodological approach.

After having performed two rounds of proteomic analyses with the three cell lines as described above, candidate proteins were identified. In total, 61 proteins were found to be differentially expressed jointly in bipyridyl-treated and resistant C1 as compared to normal C1 (Supplementary Table 2), 11 proteins were found differentially expressed in bipyridyl-treated cells only (Supplementary Table 3), and 35 proteins were found differentially expressed in resistant C1 only (Supplementary Table 4). In order to further narrow down the number of proteins specifically associated with *in vitro* metronidazole resistance and to subtract strain-specific results, metronidazole resistance was also induced in strain T1 by exposing cells to incrementally increasing metronidazole concentrations with each subculture as described before for strain C1 (Leitsch et al., 2009). High-level metronidazole resistance (1 mM metronidazole) was very easily and quickly induced in strain T1 over the duration of only two weeks. Again, three biological replicates in technical duplicates of metronidazole-susceptible and –resistant T1 were analysed by shotgun proteomics as described above for C1, and the proteins identified to be differentially expressed in resistant T1 were matched with the 35 proteins identified to be specifically differentially expressed in metronidazole-resistant C1. The shared subset containing 17 proteins (Table 2) was interpreted as specific for *in vitro* metronidazole resistance. Importantly, FR1 was also widely absent in resistant T1 which was reflected by the near-to-total loss of flavin reductase activity in cell extracts of resistant T1 (138 ± 19 nmol min^−1^ mg^−1^ as compared to 13 ± 4 nmol min^−1^ mg^−1^ in normal T1). The residual flavin reductase activity was probably exerted by FR7 which was found to be expressed in the data set of T1 but which has a much lower activity than FR1 (Leitsch et al., 2014). Further, another previously discussed candidate protein (Kulda, 1999), ferredoxin 1, was found to be strongly downregulated in resistant C1 and resistant T1 alike. Also, two isoforms of hydMDH were found to be specifically downregulated in resistant C1 and T1. The other proteins have not been associated with metronidazole resistance before and included a transcription factor (A2DLK6), a small GTPase (A2G856), proteinases (A2F7S4 and A2DMP6), and vacuolar proton pumps (A2FED9 and A2E709).

**Table 2:** Proteins consistently up- or downregulated at least 2-fold in cell lines of C1 and T1 displaying *in vitro* metronidazole resistance but not in bipyridyl-treated C1. Only proteins with a false discovery rate (FDR)-adjusted p value of <0.05 in a FDR-adjusted ANOVA with an ensuing posthoc Tukey test p < 0.01 are listed.

Protein	Uniprot ID	C1 - First	C1 - Second	T1
		Resistant vs. control	Resistant vs. control	Resistant vs. control
FR1	A2GH85	Not expressed	Not expressed	Not expressed
AP65-1 adhesin	Q27093	−6.1	−7.7	−10.3
Hydrogenosomal malic enzyme subunit B proprotein	Q27090	−3.8	−6.3	−2.9
Ferredoxin 1	A2E5A4	−21.7	−44.,3	−141.7
Cytosolic malate dehydrogenase	Q27819	−3.1	−11.2	−4.3
Peptidase T-like metallopeptidase	A2F7S4	−4.3	−8.5	−2.5
Metacaspase-like cysteine peptidase	A2DMP6	−3.1	−7	−5.5
B-box zinc finger family protein	A2DLK6	3.8	3.9	2.8
Small GTP-binding protein, putative	A2G856	2	4.5	4.9
Dynein heavy chain family protein	A2EGW8	2	5.9	5.9
Adenosylhomocysteinase	A2E342	3,1	2.3	2.2
Inosine-uridine preferring nucleoside hydrolase	A2DN71	3,1	7.3	6.6
V-type proton ATPase subunit	A2FED9	5.3	6.1	6.8
V-type proton ATPase subunit	A2E709	3.2	2.9	9.9
C2 domain containing protein	A2E8X1	4	5.8	16.2
C2 domain containing protein (Fragment)	A2GRV0	2.9	5.9	6.2
EF hand family protein	A2EAY1	2.3	4.3	3.6

Next, we wanted to compare the subset of proteins associated with *in vitro* metronidazole resistance (Table 2) with a subset of proteins specific for clinical metronidazole resistance in order to identify factors which are always associated with metronidazole resistance. To this end, a comparative proteomic analysis was performed with two metronidazole-susceptible isolates (G3 and JH31A) and two metronidazole-resistant isolates (B7268 and CDC085). All four strains had been extensively studied by us before (Leitsch et al., 2012, 2014). We considered those proteins to be associated with clinical metronidazole resistance which were up- or downregulated in both resistant isolates but in neither of the susceptible isolates. Due to a technical malfunction only two biological replicates (in technical duplicates) of the original three could be evaluated but the statistical power was, nevertheless, sufficient for use. The list of shared changes in protein expression in both resistant isolates versus both susceptible isolates was surprisingly short and contained only 15 proteins (Table 3). As expected, due to earlier observations on impaired flavin reductase activity in resistant clinical isolates (Leitsch et al., 2012, 2014), FR1 was found amongst the proteins downregulated in B7268 and CDC085.

**Table 3:** Proteins consistently up- or downregulated at least 2-fold in the metronidazole-resistant clinical isolates CDC085 and B7268 vs. the susceptible isolates JH31A and G3. Only proteins with a false discovery rate (FDR)-adjusted p value of <0.05 in a FDR-adjusted ANOVA with an ensuing posthoc Tukey test p < 0.01 are listed.

Protein	Uniprot ID	CDC085 vs. JH31A	CDC085 vs. G3	B7268 vs. JH31A	B7268 vs. G3
FR1	A2GH85	−7.7	−17.9	−3.2	−7.5
AP65-3 adhesin	Q27102	11.8	17.6	4	5.9
NADP-dependent alcohol dehydrogenase	A2F0T6	−10.7	−10	−5.1	−4.8
Coronin	Q9NFT3	72.5	113.8	56.7	88.9
Aminotransferase, classes I and II family protein	A2EIU6	−2.8	−2	−3.4	−2.4
Ornithine cyclodeaminase/mu-crystallin family protein	A2EIT3	−6.1	−4	−8.5	−5.6
PH domain containing protein	A2FGM2	3.80	6.6	4.6	7.,9
Ubiquitin family	A2EWB0	72.5	33.2	20	9.1
Surface protein	A2E3F4	2.2	10.4	2.4	11.3
EF hand family protein	A2FQM2	7.2	3.8	4.5	2.4
Transcription elongation factor 1 homolog	A2FQ96	8	11.2	4.5	6.3
Mannose-6-phosphate isomerase	A2E1E3	−4.4	−4.2	−3.4	−3.2

With all proteomic data available we subsequently performed an assessment of the validity of established notions of metronidazole resistance and matched the expression values of factors (Kulda, 1999; Leitsch, 2019) which had been previously suggested to be involved in metronidazole resistance in all strains studied (Table 4). These included PFOR, hydrogenosomal malate dehydrogenase, ferredoxins, FR1, and nitroreductases 4 and 6 (Pal et al., 2009). The latter two had been previously identified as possibly associated with metronidazole resistance (Paulish-Miller et al., 2014). Thioredoxin reductase was omitted because it is known that its expression level in resistant C1 is unchanged despite being widely inactive (Leitsch et al., 2009). PFOR was downregulated in bipyridyl-treated C1 as well as resistant C1 and resistant T1. Although the extent of downregulation was more pronounced in the resistant cell lines, PFOR levels in bipyridyl-treated cells were still very low without having any impact on metronidazole susceptibility (Table 1). Similarly, bipyridyl treatment and metronidazole resistance was accompanied by the downregulation of several isoforms of hydrogenosomal malate dehydrogenase. The extent of downregulation was either larger in bipyridyl-treated cells (Q27234 and Q27102) or smaller (Q27090 and Q27093). Of the ferredoxins, ferredoxin 1 was much stronger downregulated in the resistant C1 and T1 cell lines than in bipyridyl-treated C1. Ferredoxin 2 was absent in bipyridyl-treated C1 and in the resistant cell lines. The other ferredoxins were far less affected, with ferredoxin 3, 6, and 7 not significantly affected at all. Ferredoxin 5 was not found in either sample. Finally, FR1 was specifically absent in the metronidazole resistant cell lines of C1 and T1. In the clinical metronidazole-resistant strains B7268 and CDC085 neither of these factors except for FR1 were downregulated (Table 4). Some isoforms of hydrogenosomal malate dehydrogenase were even more strongly expressed as compared to metronidazole-susceptible G3 and JH31A. This is in line with earlier observations that clinical metronidazole-resistant strains have a normal hydrogenosomal metabolism and unimpaired PFOR activity (Müller and Gorrell, 1983). Nitroreductases 4 and 7 were not found in all strains but were present in C1. Although these nitroreductases were indeed found to be upregulated in expression in the resistant cell line, this was also found to be the case in bypiridyl-treated C1. We therefore conclude that levels of Ntr4 and 7 respond to intracellular iron levels and are not causally linked to metronidazole resistance. Also a selection of proteins which had been found to be possibly linked to metronidazole resistance in another study (Bradic et al., 2017), were analysed (Supplementary Table 5). Of 30 proteins listed by Bradic et al. we found 15 in our data sets, for which, however, no causal relation to metronidazole could be identified.

**Table 4:**
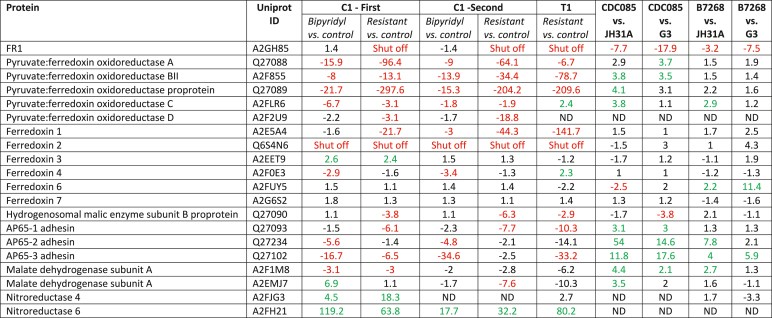
Comparison of the protein levels of selected factors previously proposed to be involved in metronidazole resistance. Red indicates downregulation by at least factor 2 with a false discovery rate (FDR)-adjusted p value of <0.05 in a FDR-adjusted ANOVA and an ensuing posthoc Tukey test p < 0.01, green indicates upregulation by at least factor 2. ND, not detected in sample. The metronidazole resistant clinical isolates are indicated in red.

### 3.2 Clinical metronidazole-resistant strains are much more tolerant to metronidazole than susceptible isolates also under anaerobic conditions

According to the widely held notion of metronidazole resistance in *T*. *vaginalis*, clinical metronidazole-resistant strains are believed to be susceptible to metronidazole under anaerobic conditions because drug activation pathways, e.g. PFOR, remain intact. In the presence of oxygen, however, metronidazole reduction is thought to be inhibited by elevated concentrations of intracellular oxygen, brought about by impaired oxygen scavenging mechanisms. FR1 has been considered an oxygen scavenging enzyme and its downregulation in metronidazole-resistant isolates is therefore congruent with this notion. However, recent findings suggest that FR1 is not quantitatively relevant for oxygen scavenging (Gehl et al., 2021) and our proteomic analysis did not reveal any other candidate enzyme potentially involved in oxygen scavenging (Table 3). Also conflicting with said notion of clinical metronidazole resistance is the previously made observation that strain B7268 exhibits a clearly reduced susceptibility to metronidazole under anaerobic conditions (Upcroft and Upcroft, 2001). We therefore wanted to re-evaluate the proposed dependence of clinical metronidazole resistance on oxygen levels and performed drug assays with all strains used in this study under anaerobic and microaerobic conditions (Lam et al., 2023). The minimal inhibitory concentration (MIC) was defined as the lowest concentration at which no motile parasites could be detected after 48 h of incubation. As shown in Table 5, metronidazole-susceptible *T*. *vaginalis* strains were overall equally susceptible to metronidazole under microaerobic and anaerobic conditions. As expected, the resistant clinical isolates were highly resistant to metronidazole under microaerobic conditions. However, also under anaerobic conditions, the resistant strains CDC085 and B7268 displayed a strongly reduced susceptibility to metronidazole. In case of B7268, at least some cells remained viable after exposure to very high metronidazole concentrations for 48 h. Further, we could routinely subculture B7268 in the presence of 50 μM metronidazole, a concentration normally lethal to *T*. *vaginalis* within several hours, without hampering growth noticeably. When subculturing CDC085 in the presence of 50 μM metronidazole, most cells died within 24 h, but growth of the remaining survivors resumed after two to three days. These results are clearly at variance with the current notion that clinical metronidazole-resistant strains are normally susceptible under anaerobic conditions and demonstrate that *T*. *vaginalis* can also display metronidazole resistance while featuring functional hydrogenosomal pathways.

**Table 5:** MICs of *T*. *vaginalis* strains/cell lines under anaerobic and microaerobic conditions. The values for C1 anaerobic were taken from Table 1. The resistant cell line of T1 was not viable under microaerophilic conditions. MICs were measured in all strains in at least three independent experiments in technical duplicates.

Strain	MIC anaerobic [μM]					MIC microaerophilic [μM]				
	Exp.1	Exp. 2	Exp. 3	Exp. 4	Exp. 5	Exp.1	Exp. 2	Exp. 3	Exp. 4	Exp. 5
C1	2.342	9.375	9.375	9.375		4.687	9.37	9.375	4.68	9.37
C1 *iv* res	2400	2400				2400				
T1	18.75	18.75	4.687			18.75	18.75	18.75	18.75	
T1 *iv* res	2400	1200	2400			/	/	/		
JH31A	9.375	18.75	9.375			2.342	2.342	4.687		
G3	18.75	18.75	9.37	4.687		4.687	9.375	9.375	9.375	18.75
CDC085	75	75	150			2400	2400	2400	2400	
B7268	600	1200	1200	600	300	2400	4800	2400	2400	

### 3.3 FR1 is a ferric iron-reducing enzyme

**Fig. 2:**
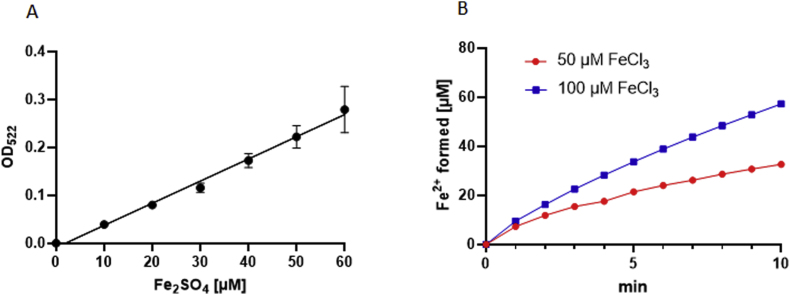
Ferric iron reductase activity of FR1. FR1 activity was measured by determining the formation of ferrous iron (Fe^2+^) from ferric iron (Fe^3+^) through the quantification of complex formation of Fe^2+^ with bipyridyl. Iron-bipyridyl complexes absorb strongly at λ_522_. The absorption values of known concentrations (10–60 μM) of ferrous iron sulphate with bipyridyl were used to make a calibration curve (A). Subsequently, the absorption values obtained with FR1 in the presence of 50 μM or 100 μM ferric iron chloride (FeCl_3_) were matched against the calibration curve (B).

The results presented above necessitated a thorough reconsideration of the mechanism of metronidazole resistance because they questioned the established model of metronidazole activation and the proposed dichotomy of *in vitro* and clinical resistance. Importantly, FR1 is the only factor consistently down-regulated in metronidazole-resistant *T*. *vaginalis* (Table 2, Table 3). However, its previously proposed function as an oxygen scavenging enzyme was not supported by measurements in live *T*. *vaginalis* (Gehl et al., 2021), and since the product of the FR1-catalyzed reduction of oxygen is harmful hydrogen peroxide (Leitsch et al., 2014), we considered it unlikely that oxygen is the primary substrate of the enzyme. We speculated that FR1 could act as a ferric iron reductase by reducing FMN which, in turn, transfers an electron to ferric iron to produce ferrous iron (Schröder et al., 2003). Indeed, when we incubated FR1 together with NADPH, FeCl_3_, FMN, and bipyridyl in cuvettes sealed with parafilm, we could measure the build-up of bipyridyl-Fe^2+^ complexes at λ_522_ (Fig. 2). The speed of the build-up was dependent on, and limited by the concentration of FeCl_3_ added. The omission of FR1, NADPH or FMN prevented the formation of bipyridyl-Fe^2+^ complexes.

### 3.4 Iron-catalyzed non-enzymatic activation of metronidazole leads to large-scale damage to cellular proteins

**Fig. 3:**
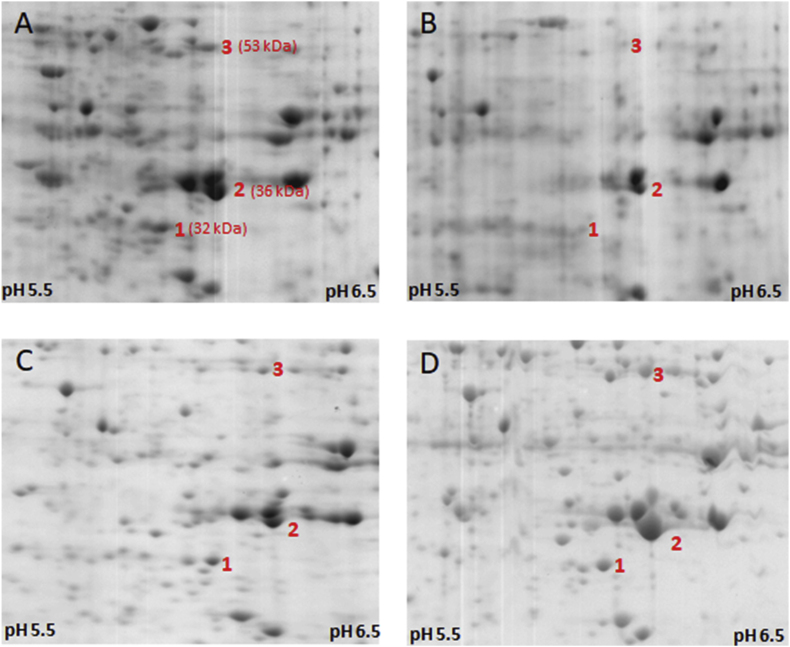
Damage to proteins in *T*. *vaginalis* C1 cell extracts. Cell extracts were incubated at RT for 30 min with NADPH (4 mM), FMN (20 μM), and 500 μM FeSO_4_ either in absence (A) or presence (B) of 1 mM metronidazole. Afterwards, proteins were precipitated with TCA and resolubilized in 2DE buffer to perform 2D gel electrophoresis. The gels were Coomassie-stained and the damage to proteins assessed by comparing the protein profiles (pH range 5–8). When cells had been preincubated with 10 μM diphenyleneiodonium (DPI) prior to preparation of the cell extract, the damage inflicted by metronidazole was greatly reduced (C). When iron was omitted and 300 μM deferoxamine (DFO) was added to cell extracts the damage inflicted by metronidazole was minimal (D). The central sections of the 2D gels are shown (approximately 30–60 kDa range; pH range approx. 5.5 to 6.5). For orientation, three proteins identified in an earlier study (Leitsch et al., 2009, 2012) are indicated: 1, thioredoxin reductase (Uniprot ID: A0A8U0WQ27); 2, cytosolic malate dehydrogenase (Uniprot ID: Q27819); 3, enolase (Uniprot ID: A2E269). The respective sizes are given in brackets. The respective entire gel images are shown in Supplementary Fig. 1.

The finding that FR1 is a ferric iron reductase led us to assume that FR1 could indeed be involved in metronidazole activation. According to a previous report, ferrous iron and cysteine can react with metronidazole to induce the formation of metronidazole radicals (Willson and Searle, 1975). Ferrous iron quickly complexes with cysteine and subsequently binds metronidazole to form a drug-iron-cysteine complex, ultimately leading to the formation of metronidazole radicals. This mechanism has so far only been demonstrated in chemical experiments but not considered in the research on metronidazole activation *in vivo*. In order to test if such mechanism could indeed apply in *T*. *vaginalis* we produced hydrogenosome- and lysosome-free extracts of *T*. *vaginalis* C1 cells by centrifugation and added NADPH, iron sulphate, and FMN, followed by incubation of the extracts for 30 min at room temperature either in the presence or in the absence of 1 mM metronidazole. Subsequently, 2D gel electrophoresis was performed in order to visualize damage inflicted on cellular proteins. The reaction was not performed anaerobically but we hypothesized that FR1 in the extract would quickly remove the oxygen and produce hydrogen peroxide. Indeed, within 1–2 min the reaction turned from yellow to colourless, indicating that FMN was now present in the reduced state and no further oxidation by oxygen occurred. Interestingly, however, no complete bleaching of FMN was observed in the sample with metronidazole, indicating that FMN was constantly re-oxidized. When we subsequently performed 2D gel electrophoresis with the thus treated cell extract, we found a large proportion of the proteins to be damaged (Fig. 3A and B) in the metronidazole-treated sample.

From one of our earlier studies, we knew that the flavin inhibitor diphenyleneiodonium (DPI) renders *T*. *vaginalis* completely insensitive to metronidazole and reduces activity of FR1 by about 75% (Leitsch et al., 2010). We wanted to test if preincubation of the cells with DPI prior to the preparation of extracts would reduce the damage to proteins as described above. This was indeed found to be the case as DPI widely suppressed the damage to proteins as observed before (Fig. 3C).

Next, we directly tested the importance of intracellular iron for this reaction and did not add 500 μM FeSO_4_ to the reaction. Instead, we added 300 μM of the iron chelator deferoxamine (DFO) because it had been observed before in a related parasite*, Tritrichomonas foetus*, that the largest proportion of intracellular iron is bound in the cytoplasm as labile iron pool, and can be complexed using DFO (Suchan et al., 2003). We speculated that the situation in *T*. *vaginalis* would be similar. Indeed, 2D gel electrophoresis showed that the damage to the cellular proteins was greatly reduced upon addition of deferoxamine (Fig. 3D). It is interesting to note that as compared to the DFO-treated sample, some protein damage could be also observed in the sample without metronidazole, arguably caused by hydroxyl radicals formed in the Fenton reaction from hydrogen peroxide and ferrous iron (Fig. 3A vs. 3D).

**Fig. 4:**
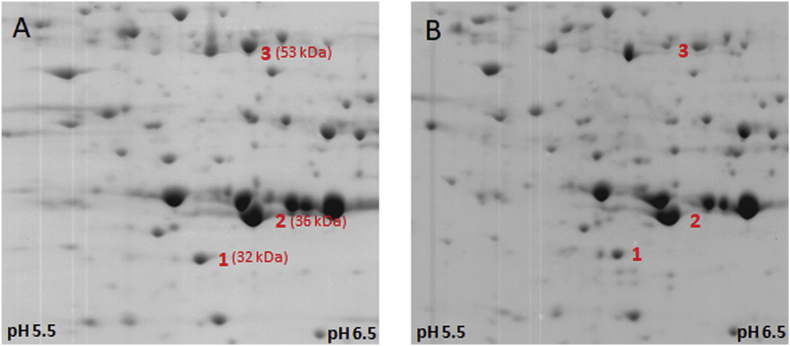
No damage to proteins can be observed when using cell extracts of metronidazole-resistant *T*. *vaginalis* B7268. Cell extracts of B7268 were prepared as described for C1 (Fig. 3) and incubated either in absence (A) or presence (B) of 1 mM metronidazole. No damage to proteins could be observed on 2D gels. The central sections of the 2D gels are shown (approximately 30–60 kDa range; pH rage approx. 5.5 to 6.5). For orientation, three proteins identified in an earlier study (Leitsch et al., 2009, 2012) are indicated: 1, thioredoxin reductase (Uniprot ID: A0A8U0WQ27); 2, cytosolic malate dehydrogenase (Uniprot ID: Q27819); 3, enolase (Uniprot ID: A2E269). The respective sizes are given in brackets. The respective entire gel images are shown in Supplementary Fig. 1.

Finally, we used extracts of metronidazole-resistant B7268 in an identical setup. As expected, very little to no damage to proteins was observed in the 2D gels, regardless of whether metronidazole had been added or not (Fig. 4).

## 4 Discussion

### *4.1* *The herein performed comparative proteomic analysis shows that the currently held notion of metronidazole resistance in* T*.* vaginalis *is inaccurate*

According to the widely accepted model of metronidazole resistance in *T*. *vaginalis* (Kulda, 1999; Leitsch, 2019) two different types exist which are fundamentally different: anaerobic or *in vitro* resistance, and aerobic or clinical resistance. The first is only observed in laboratory strains after extended exposure to sublethal doses of metronidazole and also manifests under anaerobic conditions. The second occurs in *T*. *vaginalis* strains isolated from patients who are refractory to metronidazole treatment. In these strains, metronidazole resistance was proposed to manifest only in the presence of oxygen (Leitsch, 2019). Since *T*. *vaginalis* encounters oxygen, albeit at reduced levels, in its natural habitat, the human urogenital tract (Ellis et al., 1992), this is indeed consistent with the observed treatment failures.

Anaerobic resistance was proposed to be caused by the loss of metronidazole activating enzymes. Ferredoxins were proposed as the responsible factors because they have a very low redox potential and can, therefore, transfer electrons to the nitro group of metronidazole (Moreno et al., 1983). Ferredoxins in *T*. *vaginalis* are located in the hydrogenosome, and two hydrogenosomal enzymes catalyse the reduction of ferredoxins (Kulda, 1999; Hrdy et al., 2005): PFOR and, indirectly via NADH dehydrogenase, hydrogenosomal malate dehydrogenase (hydMDH). Both, PFOR and hydMDH, were found to be downregulated in strains displaying *in vitro* resistance (Čerkasovová et al., 1984; Hrdy et al., 2005). However, it was also observed that sequestration of intracellular iron with bipyridyl leads to a near-to-total downregulation of PFOR and hydMDH without rendering the cells resistant to metronidazole (Leitsch et al., 2009). This was again confirmed in the present work (Table 1). Ferredoxin 1 was the only hydrogenosomal factor to be strongly downregulated in resistant C1 and T1 cells, but not in bipyridyl-treated C1 cells (Table 4). In an earlier study, however, it was shown that a knock-out of the respective gene did not alter the susceptibility to metronidazole (Land et al., 2004). It was offered as an explanation that the loss of ferredoxin 1 could be compensated by the other ferredoxins. Still, with the exception of ferredoxin 2 which was not expressed in resistant and bipyridyl-treated cells (Table 4), metronidazole resistance did not alter the expression of ferredoxins 3, 4, 6, and 7 (ferredoxin 5 was not detected in any sample) to a significant extent, arguing against an involvement in metronidazole reduction. Further, strain B7268, which displays considerable metronidazole resistance also in the absence of oxygen, expresses the discussed hydrogenosomal proteins, including ferredoxin 1, to a normal extent (Table 4). In summary, we deem this evidence sufficiently conclusive to constitute a rebuttal of the hypothesis of hydrogenosomal metronidazole activation.

The comparative proteomic analysis gave a list of only 17 proteins which were consistently differentially expressed in resistant C1 (2 rounds of analysis) and resistant T1 as compared to their susceptible parental strains and to bipyridyl-treated C1 (Table 2). In addition to already known candidates, i.e. hydMDH, FR1 and ferredoxin 1 (all of which downregulated), the list contained an three downregulated proteins, i.e. an isoform of cytosolic malate dehydrogenase (Q27819) and two proteases (A2F7S4 and A2DMP6), as well as ten upregulated proteins: a transcription factor (A2DLK6), a Ras-type GTPase (A2G856), two vacuolar ATPases (A2FED9 and A2E709), a calmodulin-like protein (A2EAY1), two transmembrane proteins of unknown function (A2E8X1 and A2GRV0), an isoform of dynein (A2EGW8), adenosylhomocysteinase (A2E342), and a nucleoside hydrolase (A2DN71). Further work is necessary before speculating on the roles of these factors in the development of *in vitro* metronidazole resistance because the changes could be either causative of resistance or only physiological adaptations to the fundamentally altered metabolism in these cells.

When matching alterations in protein expression in *in vitro* resistant C1 and T1 and the two clinical metronidazole-resistant strains CDC085 and B7268, we found downregulation of FR1 to be the only shared trait of all strains regarding protein expression. This suggested that the role of FR1 in metronidazole resistance might be even greater than previously suggested by the observation that episomally expressed FR1 can reconstitute metronidazole susceptibility in B7268 (Leitsch et al., 2014). Recently, the supposed role of FR1 as an oxygen scavenging enzyme was contested when a C1 cell line with early-stage *in vitro* resistance displayed an 80% downregulation of FR1 without affecting the oxygen scavenging capacity (Gehl et al., 2021). These findings prompted us to re-evaluate the function of FR1 and we discovered that in the parasite it might function as a ferric iron reductase rather than as an oxygen scavenging enzyme (Fig. 2).

### 4.2 A novel model of metronidazole activation and resistance

A model of non-enzymatic reduction of metronidazole by ferrous iron and cysteine in the absence of oxygen was proposed nearly 50 years ago (Willson and Searle, 1975), but has so far not been considered as a major drug activating pathway in living anaerobes/microaerophiles. According to this model, ferrous iron binds to cysteine (either as free amino acid or as a constituent of proteins) forming a complex of two cysteine ligands and one ferrous iron ion. Oxygen can oxidize the ferrous iron in this complex to unreactive ferric iron, resulting in the formation of superoxide radical anions. Alternatively, this complex can be bound by metronidazole or other 5-nitroimidazoles. This ferrous iron-cysteine-metronidazole complex is then resolved through a reaction with cysteine, which results in the formation of metronidazole nitro radical anions and of cysteine thiyl radicals. The resulting radicals can then react with each other or with nucleophilic centers in their direct vicinity. If the reaction occurred at protein-bound cysteines it is reasonable to assume that the peptide backbone is being attacked leading to the fragmentation of proteins (Stadtman, 2006; Kristensen et al., 2021). We observed very marked protein fragmentation when incubating *T*. *vaginalis* C1 cell extracts with metronidazole, NADPH, ferrous iron, and FMN (Fig. 3A). Some fragmentation, however, was also caused in the absence of metronidazole, presumably caused by hydroxyl radicals generated through reaction of hydrogen peroxide with ferrous iron (Fenton chemistry). This fragmentation was quenched (Fig. 3C and D) when proteins were stripped of iron using deferoxamine, and when cells had been preincubated with DPI, a flavin inhibitor which reduces FR1 activity to about 25% (Leitsch et al., 2010). In cell extracts of metronidazole-resistant B7268, very little to no protein fragmentation was observed (Fig. 4).

**Fig. 5:**
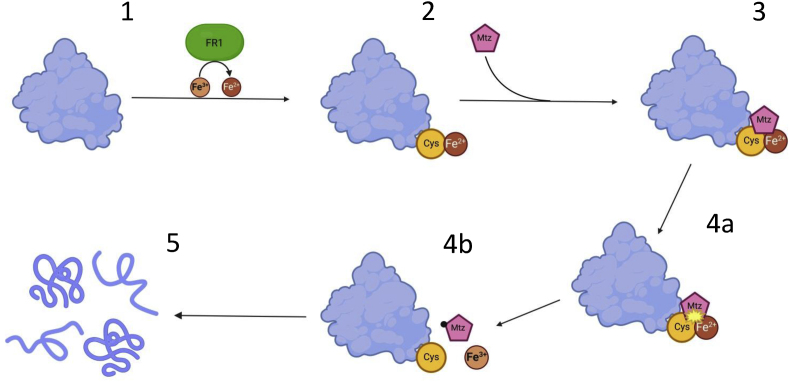
Novel model of metronidazole activation and resistance. In normal *T*. *vaginalis* cells FR1 reduces ferric iron (Fe^3+^) to ferrous iron (Fe^2+^) [1] which then binds to cysteines of cytoplasmic proteins to form iron-cysteine complexes [2], constituting the labile iron pool. When metronidazole enters the cells [3] it forms a triple complex with these complexes leading to the formation of metronidazole radicals upon their resolution (Willson and Searle, 1975). The metronidazole radicals react with the nearby protein leading to the formation of adducts and to breaks in the peptide backbone [4], thereby destroying the proteins. In metronidazole-resistant cells, FR1 is not, or only weakly active and levels of ferrous iron are low. Consequently, the cysteines in cytoplasmic proteins are not bound by ferrous iron and not primed for attack by metronidazole. Created with BioRender.com.

We propose (Fig. 5) that *T*. *vaginalis*, and possibly other anaerobes and microaerophiles, are rendered susceptible to metronidazole because they exhibit large amounts of iron on their cellular proteins as a labile iron pool. In the related parasite *T. foetus* the labile iron pool had before been found to be very large (Suchan et al., 2003) which contrasts with the situation to aerobic organisms where the labile iron pool is small due to its high reactivity with oxygen and reactive oxygen species. Further, the labile iron pool in *T*. *foetus* is mainly (>85%) constituted by cytoplasmic molecules larger than 5 kDa which is strongly indicative of a major involvement of cytoplasmic proteins. Iron binding enables the formation of radicals directly at these the proteins, rendering them prone to damage caused by the radicals formed. FR1 in *T*. *vaginalis* acts as a ferric iron reductase and thereby provides the ferrous iron which then can bind to the proteins rendering them sensitive to a metronidazole attack. In the absence of FR1 activity, less iron is complexed to proteins, especially in the presence of oxygen which re-oxidizes the protein-bound iron (Willson and Searle, 1975). This might explain why the loss of FR1 activity manifests more clearly as metronidazole resistance in the presence of oxygen. Under anaerobic conditions, and therefore undisturbed by oxygen, the reduction of ferric iron to ferrous iron might also be catalyzed more slowly by other factors, and the downsizing of the intracellular iron pool is presumably necessary to prevent loading of proteins with iron. The reduction of iron uptake, in turn, must lead to the downregulation or the loss of function of iron-dependent enzymes, most prominently of those hydrogenosomal enzymes which were previously believed to be responsible for metronidazole activation. Such downregulation can also be observed in bipyridyl-treated cells (Supplementary Table 2), but since FR1 is normally active, residual intracellular iron is sufficient to load proteins with iron and to render them sensitive to metronidazole attack.

The proposed mechanism of metronidazole activation is not the only activation mechanism in *T*. *vaginalis*. The flavin enzyme TrxR, e.g., can also reduce metronidazole and the subsequent formation of adducts with metronidazole and other 5-nitroimidazoles, was shown to impair the enzymes activity (Leitsch et al., 2009). Also other enzymes which are likely to be in spatial proximity to TrxR were found to form adducts with 5-nitroimidazoles. Still, the reactivity of TrxR with 5-nitroimidazoles is slow (Leitsch et al., 2009) and the observed depletion of intracellular cysteine pools is much more likely to be caused by radicals formed as described above. Furthermore, although TrxR was shown to be inactive in metronidazole-resistant C1 (Leitsch et al., 2009), it remains fully functional in clinical metronidazole-resistant strains (Leitsch et al., 2012). Consequently, we propose that the novel mechanism of metronidazole activation as outlined above (Fig. 5) constitutes the major source of reactive metronidazole intermediates in *T*. *vaginalis*. Labile iron pools can exist in microorganisms with an anaerobic metabolism because the absence of oxygen renders them rather safe. Aerobic organisms or facultative anaerobes, however, employ a different mode of iron storage because readily available ferrous iron would render them highly sensitive to reactive oxygen species, especially to hydrogen peroxide which decays to highly toxic hydroxyl radicals when reacting with ferrous iron.

## 5 Conclusion

Our data, of course, do not preclude the existence of other resistance mechanisms. The proteomic analyses, e.g., have their limitations regarding detection limits of peptides and the variability of 2DE analyses and respective spot assignments. This is evidenced by the absence in the data sets of four MYB-like transcription factors (Supplementary Table 5) which had before been found downregulated in a transcriptomic study (Bradic et al., 2017). Also, the solubilization of proteins prior to mass spectrometry is incomplete and can lead to an underrepresentation of membrane proteins. Finally, some important alterations might not be reflected by differential protein expression. In an earlier study we showed that TrxR is normally expressed but inactive in metronidazole-resistant C1, and that activity can be restored in enzyme assays by addition of FAD which is a cofactor of the enzyme (Leitsch et al., 2009). Similar observations were also made with a zinc-dependent alcohol dehydrogenase (Leitsch et al., 2012) which was inactive in enzyme assays using cell extracts of resistant C1 unless Zn^2+^ ions were added to the mix. Future studies will therefore have to address metal concentrations in metronidazole-resistant strains and also the concentrations of important cofactors such as FAD, FMN, and NADPH.

## CRediT authorship contribution statement

**Anna-Lena Mayr:** Writing – review & editing, Methodology, Investigation. **Ana Paunkov:** Methodology, Investigation. **Karin Hummel:** Supervision, Investigation. **Ebrahim Razzazi-Fazeli:** Writing – review & editing, Supervision, Conceptualization. **David Leitsch:** Writing – original draft, Validation, Investigation, Funding acquisition, Formal analysis, Data curation, Conceptualization.
