# Supplementary material for: Comparative proteomic analysis of metronidazole-sensitive and resistant Trichomonas vaginalis suggests a novel mode of metronidazole action and resistance
Source: Int J Parasitol Drugs Drug Resist. 2024 Sep 26;26:100566. doi: 10.1016/j.ijpddr.2024.100566 (PMC11490683; doi:10.1016/j.ijpddr.2024.100566)
Supplement: Multimedia component 1 [file mmc1.pdf]

Supplementary Figure 1

Whole 2D gels of cell C1 cell extracts after incubation with NADPH, FMN, $FeSO_4$ and ± metronidazole

These figures shows the complete 2D gels of which the sections are displayed in figures 3 and 4 of the manuscript

First dimension: pH 5-8
Second dimension: 12.5% PAA

Comparison of C1 extracts either with or without metronidazole

C1 extract -metronidazole

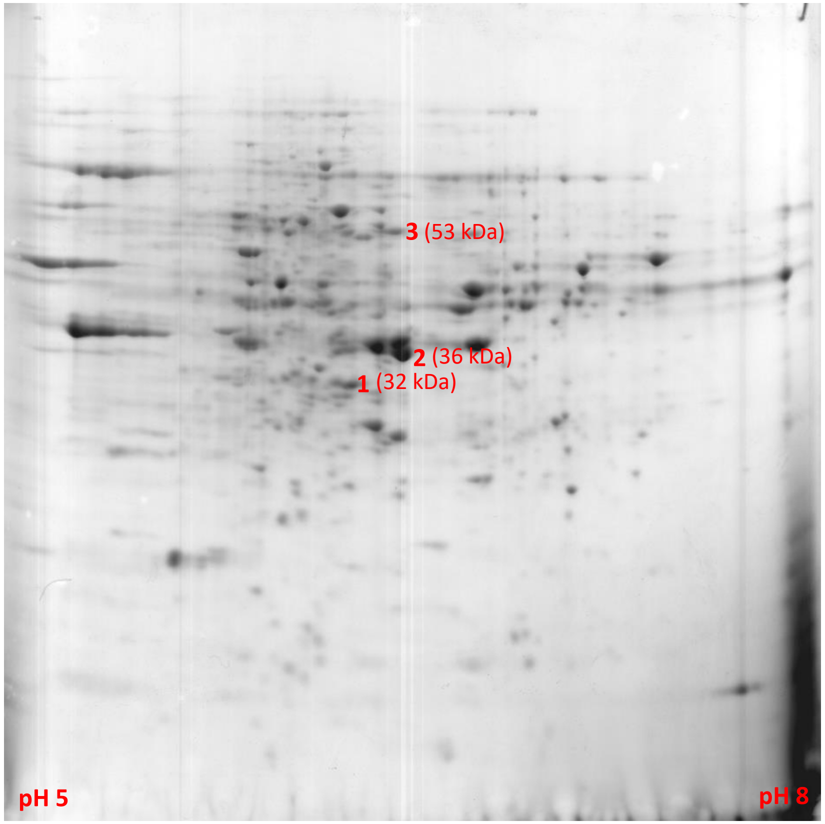

C1 extract + 1mM metronidazole

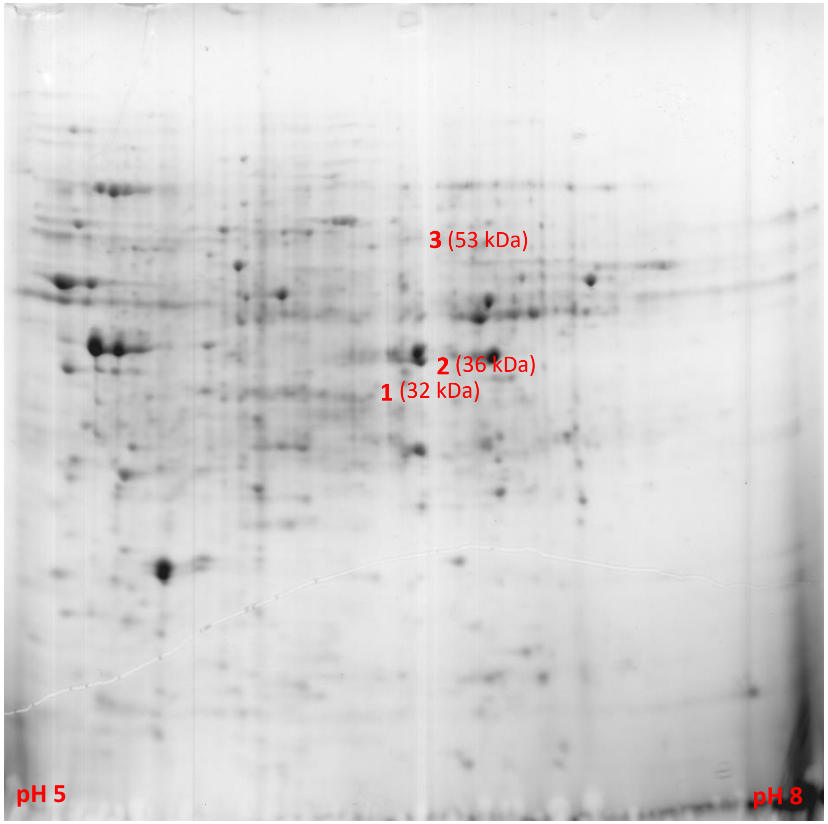

1, thioredoxin reductase (Uniprot ID: A0A8U0WQ27); 2, cytosolic malate dehydrogenase (Uniprot ID: Q27819); 3, enolase (Uniprot ID: A2E269). The respective sizes are given in brackets.

Comparison of extracts from C1 cells either preincubated with DPI or not

C1 extract + 1mM metronidazole

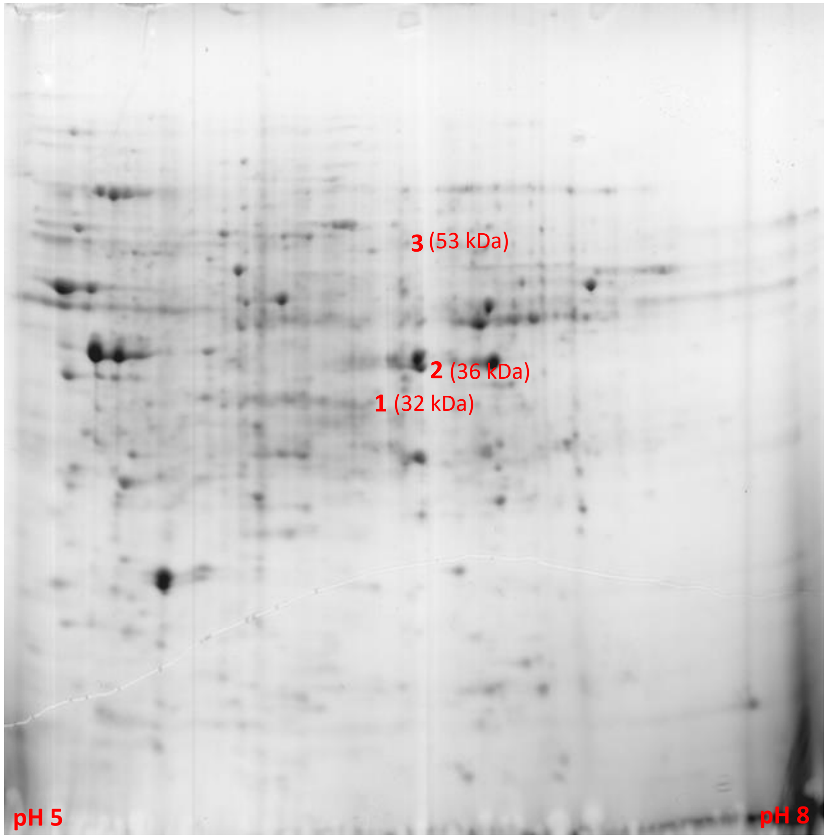

C1 extract + 1mM metronidazole
Cells preincubated with 10 µM DPI for 2h prior to lysis

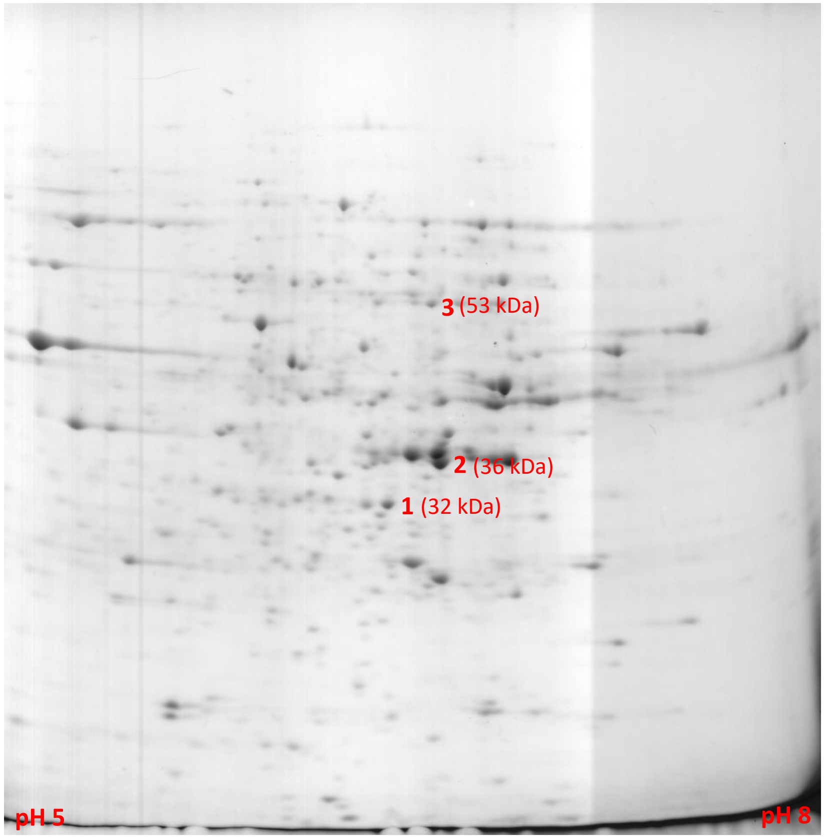

1, thioredoxin reductase (Uniprot ID: A0A8U0WQ27); 2, cytosolic malate dehydrogenase (Uniprot ID: Q27819); 3, enolase (Uniprot ID: A2E269). The respective sizes are given in brackets.

Comparison of extracts from C1 cells + metronidazole and ± DFO

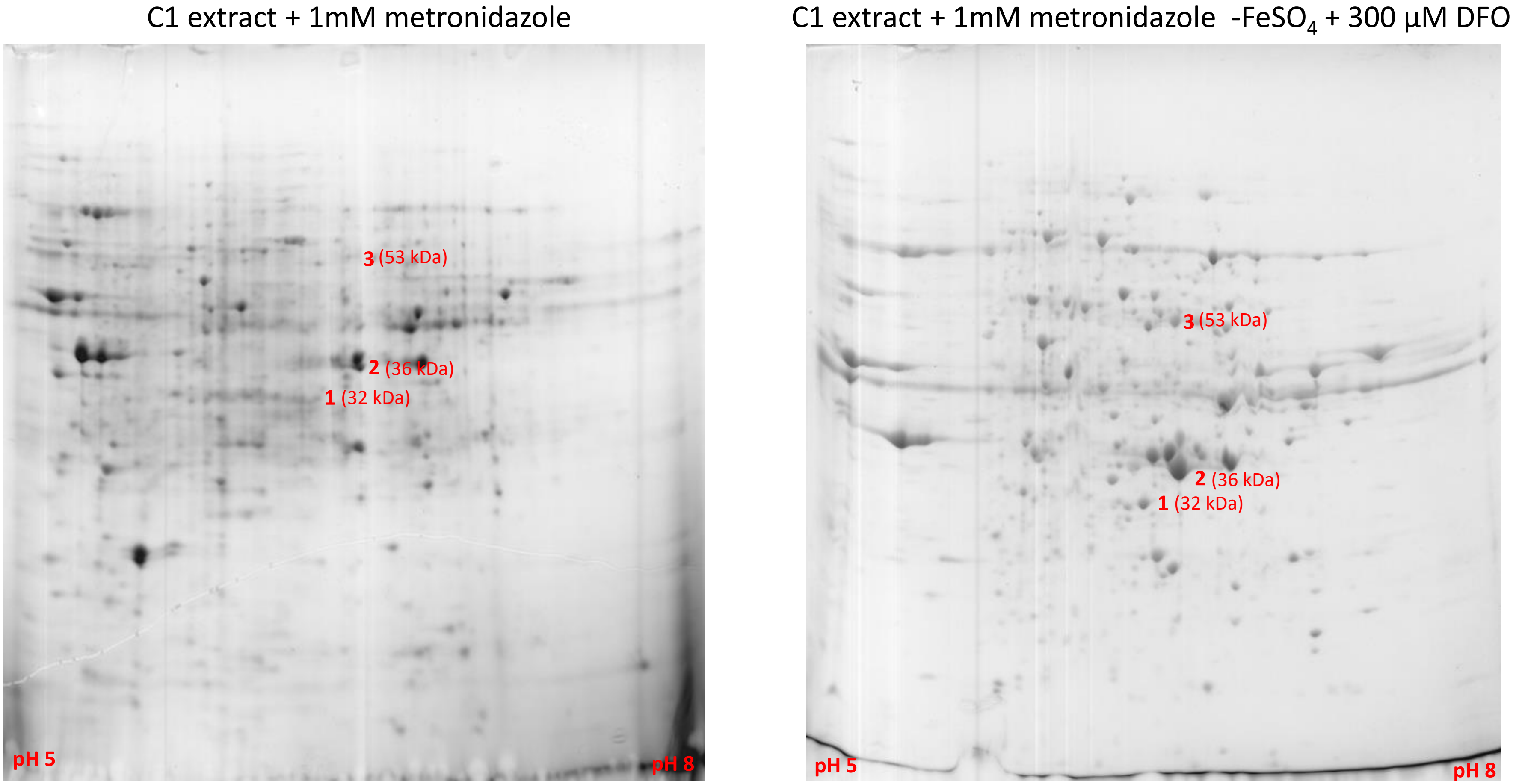

1, thioredoxin reductase (Uniprot ID: A0A8U0WQ27); 2, cytosolic malate dehydrogenase (Uniprot ID: Q27819); 3, enolase (Uniprot ID: A2E269). The respective sizes are given in brackets.

Comparison of B7268 extracts either with or without metronidazole

B7268 extract -metronidazole

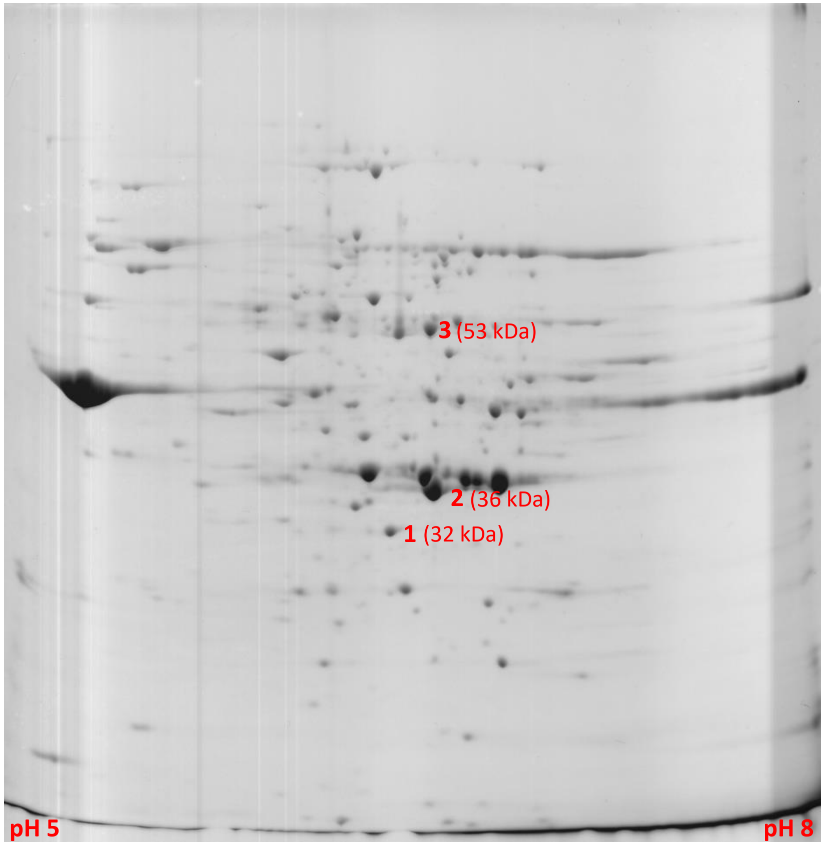

B7268 extract + 1mM metronidazole

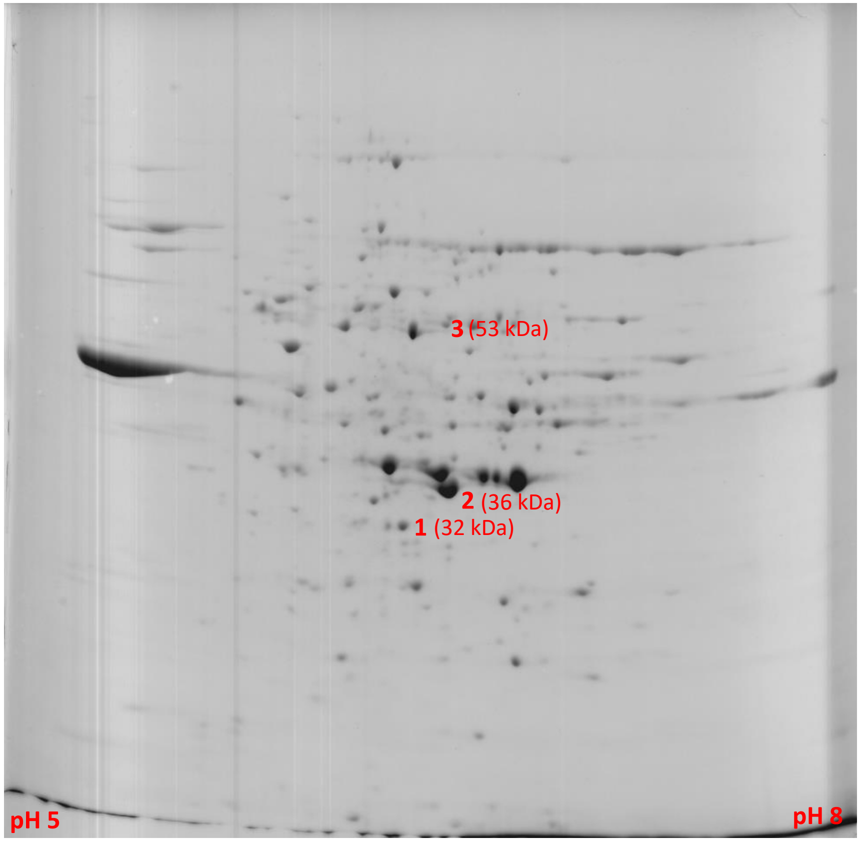

1, thioredoxin reductase (Uniprot ID: A0A8U0WQ27); 2, cytosolic malate dehydrogenase (Uniprot ID: Q27819); 3, enolase (Uniprot ID: A2E269). The respective sizes are given in brackets.

2D gels from three independent experiments showing damage to proteins by metronidazole

The extent of damage is reproducible

C1 extract + 1mM metronidazole (1)

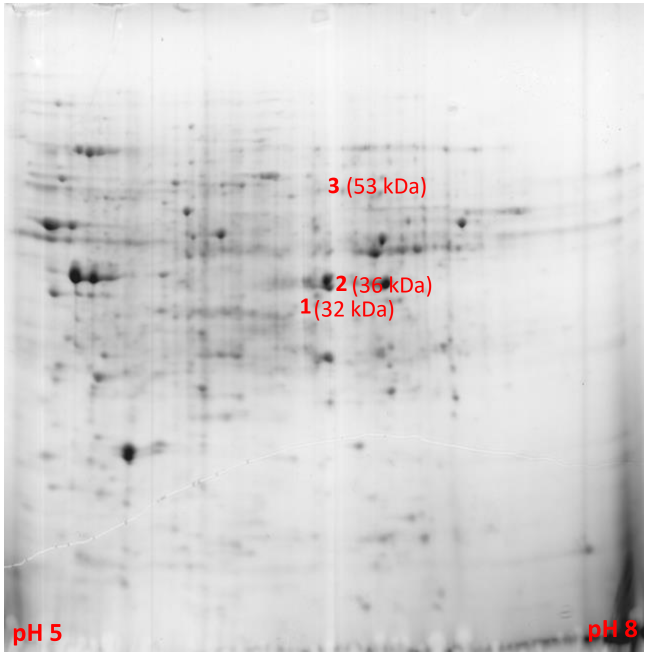

C1 extract + 1mM metronidazole (2)

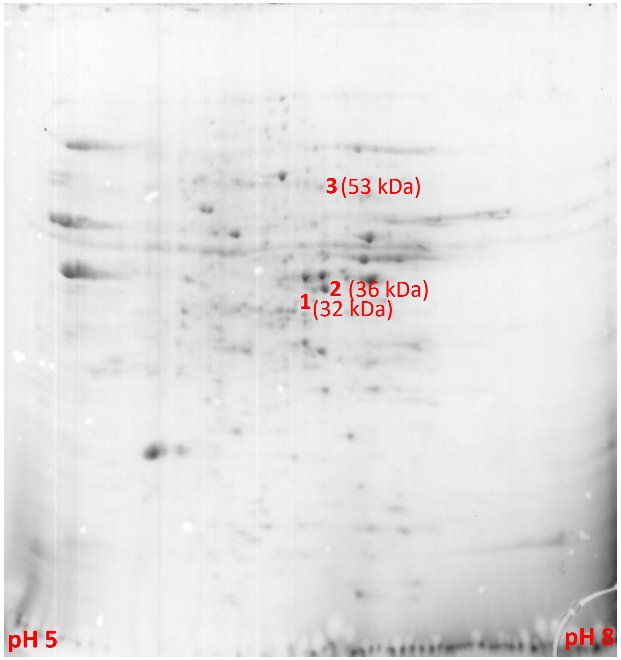

C1 extract + 1mM metronidazole (3)

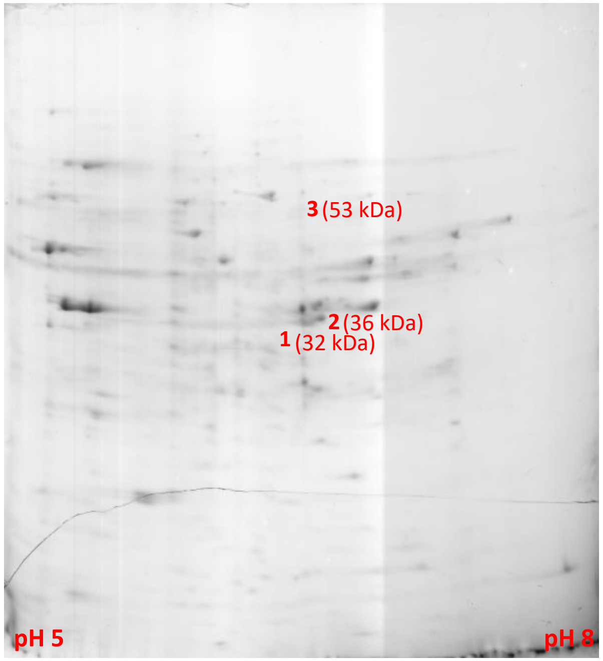

1, thioredoxin reductase (Uniprot ID: A0A8U0WQ27); 2, cytosolic malate dehydrogenase (Uniprot ID: Q27819); 3, enolase (Uniprot ID: A2E269). The respective sizes are given in brackets.
